# Convergent and divergent neural circuit architectures that support acoustic communication

**DOI:** 10.3389/fncir.2022.976789

**Published:** 2022-11-17

**Authors:** Darcy B. Kelley

**Affiliations:** Department of Biological Sciences, Columbia University, New York, NY, United States

**Keywords:** vocal, auditory, neural, circuit, communication, evolution, sex, hormones

## Abstract

Vocal communication is used across extant vertebrates, is evolutionarily ancient, and been maintained, in many lineages. Here I review the neural circuit architectures that support intraspecific acoustic signaling in representative anuran, mammalian and avian species as well as two invertebrates, fruit flies and Hawaiian crickets. I focus on hindbrain motor control motifs and their ties to respiratory circuits, expression of receptors for gonadal steroids in motor, sensory, and limbic neurons as well as divergent modalities that evoke vocal responses. Hindbrain and limbic participants in acoustic communication are highly conserved, while forebrain participants have diverged between anurans and mammals, as well as songbirds and rodents. I discuss the roles of natural and sexual selection in driving speciation, as well as exaptation of circuit elements with ancestral roles in respiration, for producing sounds and driving rhythmic vocal features. Recent technical advances in whole brain fMRI across species will enable real time imaging of acoustic signaling partners, tying auditory perception to vocal production.

## The evolution of vocal communication in tetrapod vertebrates; Introduction and overview

**Figure 1:**
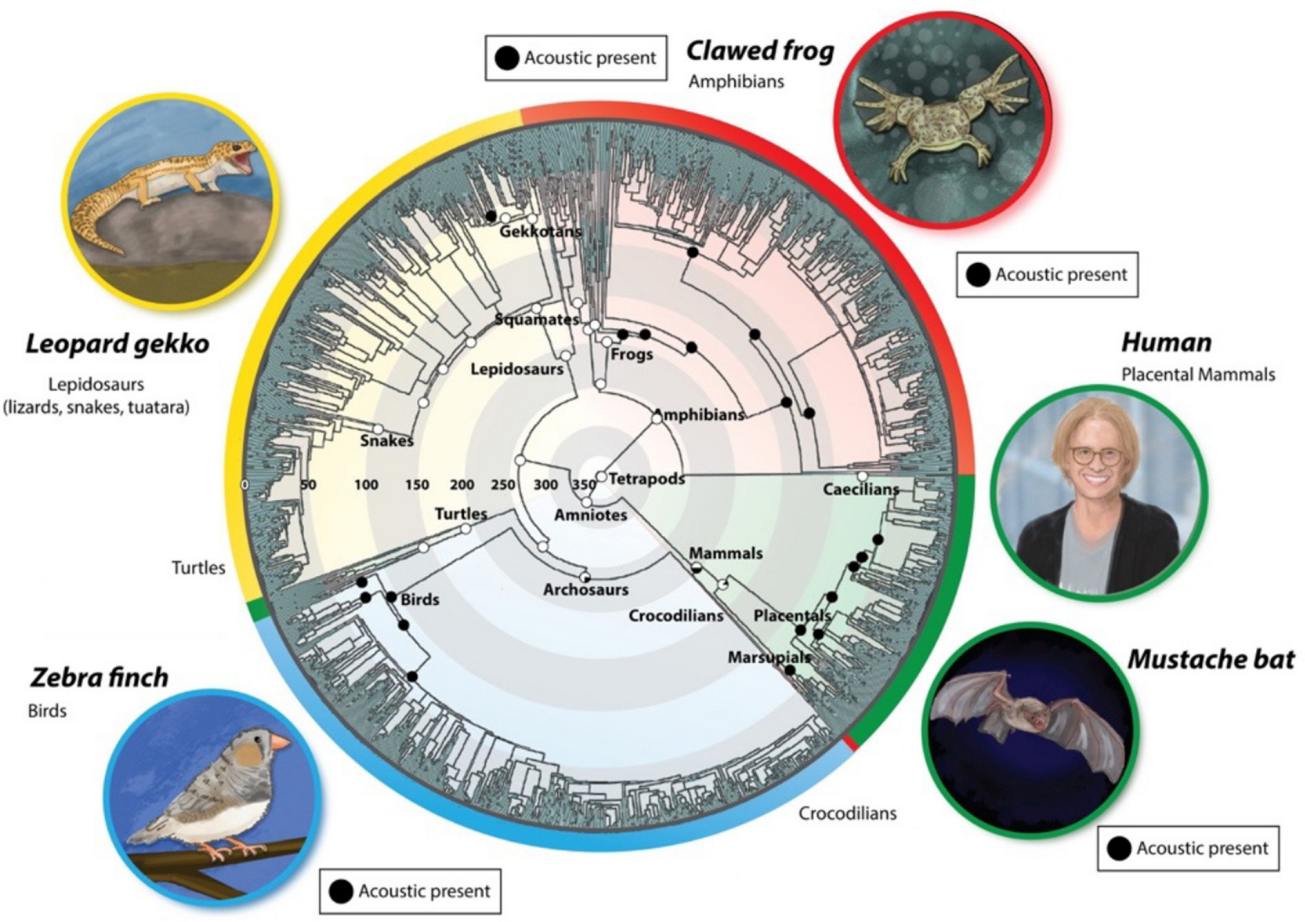
Evolution of vocal communication in tetrapods [modified from Chen and Wiens (2020)]. Extant species occupy the outer rings, color-coded according to species group (red: *amphibia*; green: *mammalia*; blue: *aves*; yellow: *reptilia*). Evolutionary time is represented radially, beginning 350 million years ago (mya) at the center and ending at the outer edge of the circle (0 mya). Within a group, related species are adjacent circumferentially. The black circles depict extant acoustic communication in taxa. Recent evidence for vocal production in turtles and Lepidosaurs supports a more ancient origin for vocal communication (Jorgewich-Cohen et al., 2022).

Acoustic communication plays an essential role in social behaviors of many species. In tetrapod vertebrates (Figure 1), both the cries of infants and the songs used in courtship are the result of neural circuit activity that drives muscles interposed between the lungs and the mouth. Sensory, CNS and motor systems that support innate, species-specific vocal communication reflect heritable genetic differences over evolutionary times scales. For example, crying is an innate behavior in infants (deaf babies cry), a key component of social interactions in our species. As human listeners misinterpret distress levels conveyed by tempo and pitch in cries from other primate species (bonobo and chimpanzee, Kelly et al., 2017), baby *Homo neanderthalis* and *sapiens* cries were probably species-specific. Courtship songs in other tetrapods are also typically innate, with only a few exceptions, most notably songbirds (Jarvis, 2019). Producing and recognizing different innate vocalizations (e.g., call types in birds; crying, sighing, laughter in *H. sapiens*) is essential for social communication (Simonyan and Horwitz, 2011; Rose et al., 2022).

Acoustic communication is ancient in tetrapods and was maintained over long periods: (Chen and Wiens, 2020; Jorgewich-Cohen et al., 2022). How did the neural circuit architectures that support the production and perception of songs evolve? Figure 1 illustrates extant species that serve as models for the neural bases of vocal communication and their evolutionary histories. This review aims to compare CNS circuits that produce and respond to innate vocalizations in these models to identify conserved and divergent features across evolutionary time scales.

**Anurans** (frogs and toads) are among the most ancient acoustic communicators, appearing in the fossil record ~270 mya (Figure 1). Within the Anura, the terrestrial Neobatrachians (e.g. *Ranids*) emerged from a world-wide extinction event and underwent massive radiations at the KT boundary (~68 mya; Feng et al., 2017). The Archebatrachians, in contrast, neither became extinct, nor radiated massively, but instead gave rise to aquatic anurans, the *Xenopodinae*, exceptionally well-represented in the fossil record (Cannatella, 2015) as well by 29 extant species (Evans et al., 2015). *Xenopus laevis* from South Africa was adopted by biologists in the 1800s for studies in experimental ethology, development, endocrinology, cell biology, and neurobiology (Wallingford, 2022). Males in all 29 species communicate vocally (Tobias et al., 2011), as do females, although one species group (A) has lost the female release call (Tobias et al., 2014).

*Xenopus* are secondarily aquatic (derived from terrestrial ancestors) and vocalizations are produced by a larynx modified for underwater sound production to produce sounds without airflow (Kwong-Brown et al., 2019). The neural circuits that support sex- specific acoustic communication have been elucidated and hindbrain neurons responsible for species-specific song rhythms identified [reviewed in Kelley et al. (2020)]. Identifying the genetic basis of the production and reception of species-specific vocal signals in *Xenopus* (clawed frogs) is our current research focus.

**Placental mammals** are also ancient acoustic communicators (~80 mya; Figure 1). The **Rodentia**—having diverged from langiomorphs (rabbits) ~85 mya—include many vocal genera (*Mus, Scotinomys, Rattus, Heterocephalus*). Mice (*Mus*) have dominated investigations of rodent ultrasonic vocalizations (USVs), due in part to the genetic advantages of specific laboratory strains. The **Chiroptera** (bats)—another highly vocal group—diverged from other mammals ~75 mya (Agnarsson et al., 2011). In some species, bats use vocalizations in both social communication and prey capture (echolocation). Among **Hominids**, the ancestor of our own species (*H. sapiens*) is recent (~200–250 kya; Vidal et al., 2022). *Homo sapiens* is the sole extant species and we do not know whether, for example, *H. neanderthalensis* spoke or sang. In all mammals, the larynx is the major organ of vocal expression. Sounds are powered by expiration and shaped acoustically by the vocal tract (Milsom et al., 2022).

**Birds** evolved from Archosaurs (dinosaurs) ~240 MYA and bird species radiated ~60 mya, again reflecting the worldwide extinction event at the K-T boundary. All extant birds communicate vocally (Figure 1), suggesting that ancestral dinosaurs sang as well. Avian behaviors were a focus of early ethologists (e.g., Lorentz and Tinbergen) and the discovery of geographical dialects in some species, provided experimental model systems for vocal motor learning. The zebra finch (Figure 1, lower left), *Taeniopygia guttata*, is currently the “lab rat” for the study of bird song neural circuits. While song control nuclei in the forebrain are not homologs of mammalian cortical areas that participate in acoustic communication, they exhibit convergent neural circuit architectures including, for example, a role for dopamine in song learning (Gadagkar et al., 2016).

I begin this review by examining neural circuit mechanisms that receive and generate species-specific *Xenopus* songs and then compare shared and divergent circuit motifs with other vocal vertebrates. As invertebrates are also prominent acoustic communicators. I conclude by comparing circuit motifs in tetrapods to acoustic communication in two invertebrates—fruit flies and Hawaiian crickets.

## Phylogeny of vocal signaling in *Xenopus*

Understanding how the nervous system generates and responds to vocal signals and how circuit architectures diverge evolutionarily ideally requires a multispecies genus that communicates vocally, in which both the neural circuits that generate vocalizations—and those that respond to socially relevant sounds—can be mapped, characterized and compared electrophysiologically and anatomically, and the underlying genetic architectures of key neurons identified. Neural circuits are constructed developmentally and thus easy access to the nervous system at all developmental stages is advantageous. These features are all prominent in *Xenopus*, the focus of our experimental studies for many decades (see Kelley et al., 2020).

**Figure 2:**
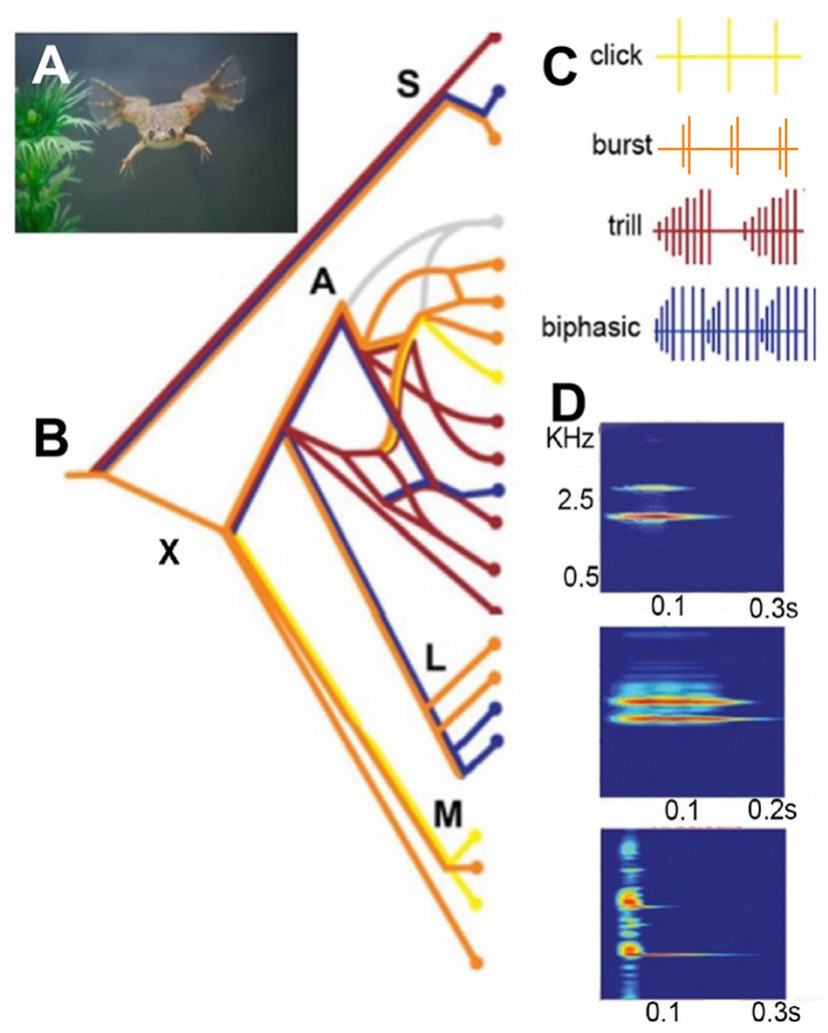
**(A)** *Xenopus* sing underwater. **(B)** A simplified phylogeny of extant species based on Evans et al. (2015). The genus *Xenopus* includes two sub-genera: *Silurana* (*S*) and *Xenopus* (*X*). *X* is made up of three species sub-groups: A, L, and M. Speciation progresses largely by hybridization (see sub-group A). *Silurana* includes the only diploid species in the genus: *X. tropicalis*, as well as two tetraploid species*: X. epitropicalis* and *X. mellotropicalis*. Ploidy levels in the sub-genus *Xenopus* ranges from tetraploid (e.g. sub-group L) to dodecaploid (sub-group A). **(C)** Male advertisement calls comprise brief sound pulses (vertical lines) in four patterns: click, burst, trill and biphasic (Tobias et al., 2011). Each species group includes multiple patterns. A parsimony analysis suggests that the ancestral call type was a burst: (**B**: orange horizontal line at far left). **(D)** A single sound pulses in male advertisement calls from three different species. Each pulse includes two dominant frequencies: DF2 (higher) and DF1 (lower) (Kwong-Brown et al., 2019). Scale bars: X-axis, sound frequency in kiloHertz (kHz). Y-axis, time in seconds (s). The combination of pattern and sound frequency is a unique species identifier (Tobias et al., 2011). Modified from Leininger and Kelley (2013).

Each species of *Xenopus* can be identified definitively from the temporal and spectral features of male advertisement calls (Figure 2B; Tobias et al., 2011; Evans et al., 2015). A major experimental advantage of *Xenopus* are the *ex vivo* preparations: the vocal organ and the brain that “sing in the dish.” The *ex vivo* larynx generates fictive songs (sound pulse patterns) in response to stimulation of attached laryngeal nerves that mimics call patterns (Figure 2); spectral features are identical to actual calls (Tobias and Kelley, 1987). The *ex vivo* brain generates fictive songs, patterned laryngeal activity nerve that matches actual vocalizations, in response to application of the neuromodulator serotonin (Rhodes et al., 2007). Spectral features are thus created within the larynx without air flow while temporal features are generated by neural circuits within the CNS (Luksch et al., 1996; Kelley et al., 2020). Application of fluorescent dextran amines to the *ex vivo* brain allows visualization of neurons within specific brain nuclei that receive auditory input as well as the auditory vocal interface and components of the vocal motor pathways, including axonal trajectories (reviewed in Kelley et al., 2020). Se*x*-specific characteristics of these neural circuits and of the larynx are due to hormone-regulated development (Zornik and Kelley, 2011). These *ex vivo* preparations allow us to experimentally determine which hormonally regulated, sexually differentiated features are organ-autonomous and which reflect brain-larynx interactions.

## How *Xenopus* communicate

**Figure 3:**
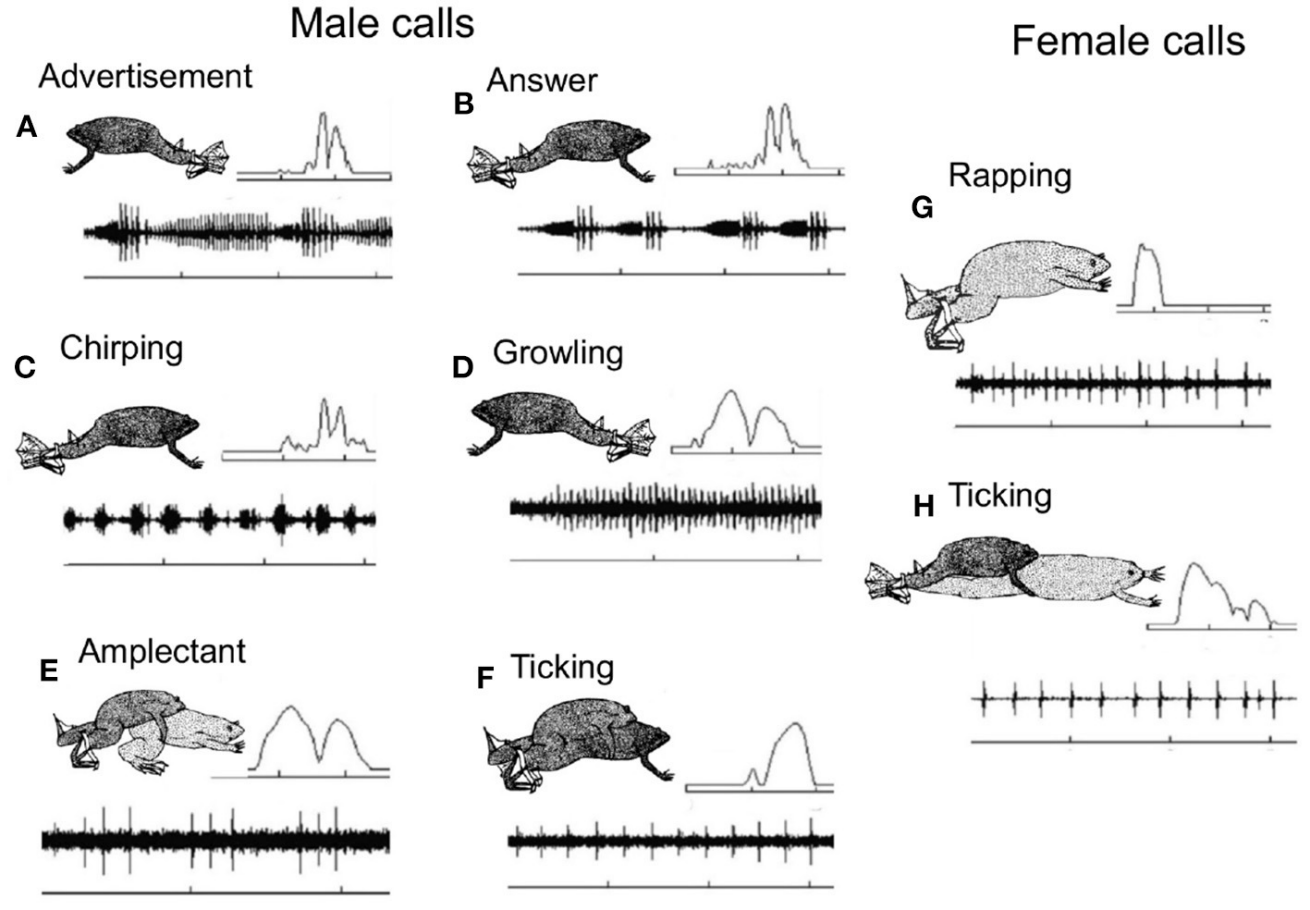
*Xenopus* preferentially inhabit turbid ponds with low visibility, so pairs of same and opposite sex *X. laevis* were also recorded in the laboratory to identify the caller. The vocal repertoire of male and female *Xenopus laevis* recorded from a pond outside of Capetown, South Africa at the onset of the breeding season (Tobias et al., 2004). The X- axis is time and the Y- axis is sound amplitude. The most frequent vocalization is the male advertisement call **(A)**, a series of slow and fast trills. Each sound pulse includes two dominant frequencies (inset, upper right. Frequency scale 1–3 kHz). Time scales: 1s except D: 0.5s. Original drawings by Barbara Goun. In response to the female advertisement call, rapping (Tobias et al., 1998; see **G**), the male produces an answer call **(B)** in which the slow trill is shortened and the amplitude modulation of the fast trill is enhanced (relative to the advertisement call. Pairs of sexually active males chirp **(C)** and growl **(D)**; one establishes vocal dominance and the other is silenced (Tobias et al., 2010). When clasping a female, males produce the amplectant call **(E)** and when a male is clasped by another male, the clasped male ticks **(F)**. Just prior to oviposition females produce the rapping call **(G)**. Rapping is an acoustic aphrodisiac for males, stimulating male answer calling, male/female duets and male approach (Tobias et al., 1998). Sexually unreceptive females tick **(H)** and extend their hind legs (Kelley and Pfaff, 1976). These highly specific vocal interactions facilitate studies of the functional roles of different brain nuclei in the context of acoustic communication.

Recordings from a South African pond across the breeding season together with laboratory studies (Tobias et al., 2004) reveal a rich vocal repertoire specific to social context and sex in *Xenopus laevis*, the most widely studied species (Figure 3).

## How *Xenopus* make sounds

In most tetrapods, sounds are powered by expiration of air from the lungs driving vibrations of the vocal folds (Ghazanfar and Rendall, 2008; Fitch and Suthers, 2016) or—in birds—of the internal tympaniform membranes (Elemans, 2014). In mice, for example, ultrasonic vocalizations are produced by high velocity air jets that power vocal fold vibrations (Håkansson et al., 2022). CNS respiratory and vocal circuitry must thus be closely linked.

**Figure 4:**
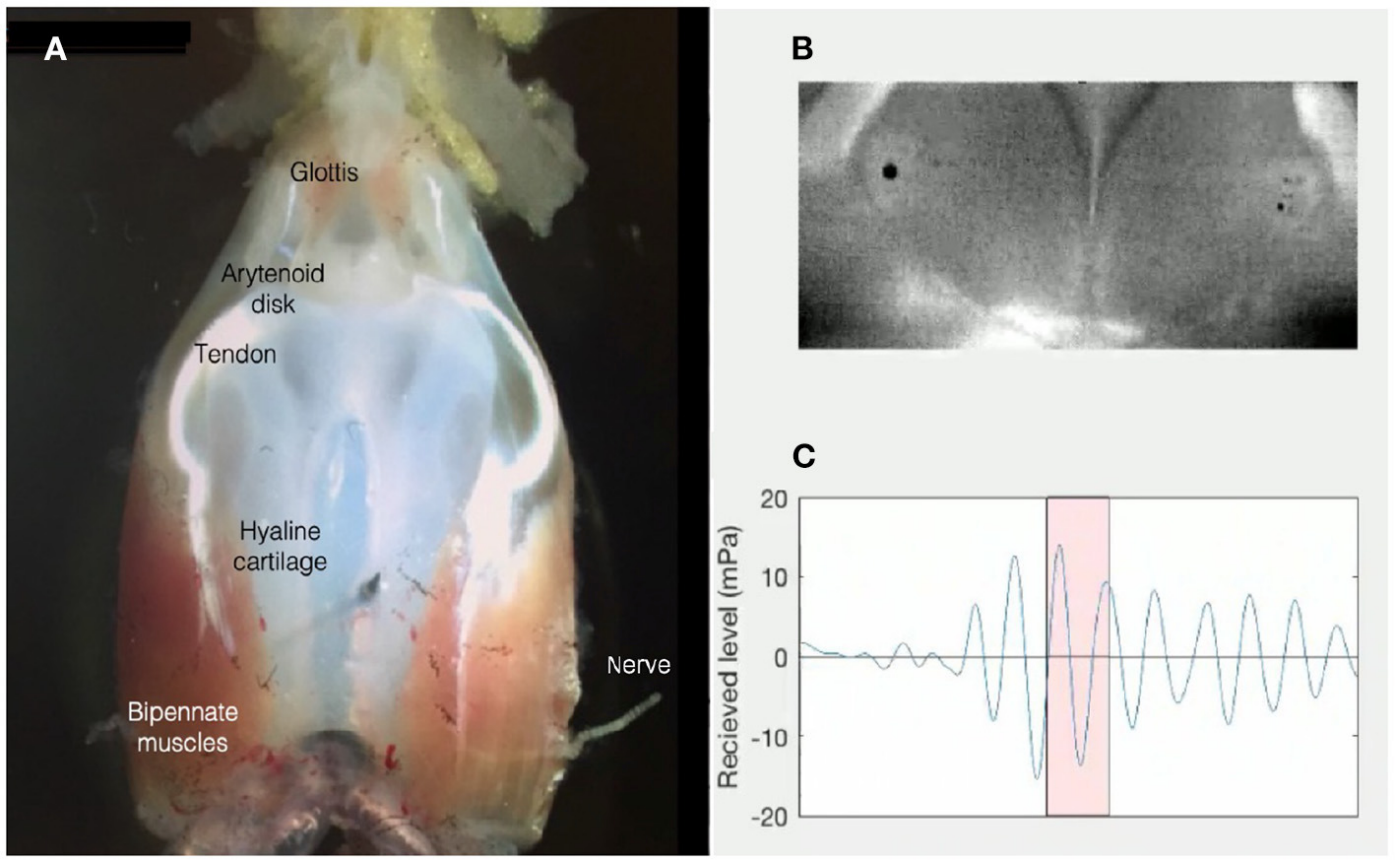
**(A)** The *ex vivo* larynx of an adult male *X. laevis* is composed of a frame of hyaline cartilage, flanked by bipennate muscles that insert anteriorly into the sound-producing, paired arytenoid disks via a tendon. The larynx is attached to the lungs posteriorly. The anterior opening into the buccal cavity is gated by the glottis. The laryngeal nerve includes axons of laryngeal and glottal motor neurons located in hindbrain: Nucleus Ambiguus. **(B)** Opening and closing of the arytenoid disks during high-speed video recordings reveal that a sound pulse **(C)** results when disk opening reaches a critical velocity [Figure modified from Kwong-Brown et al. (2019)]. Sounds produced by the *ex vivo* larynx are also audible in air (Tobias and Kelley, 1987).

However, the ability of the *ex vivo Xenopus* larynx to create sounds in the absence of air flow from the lungs, together with observations that these sounds are not shifted in frequency by heliox (Yager, 1982; Kwong-Brown et al., 2019), suggested that *Xenopus* do not use air flow to power their underwater songs. Instead, sounds are created by rapid separation of intra-laryngeal arytenoid cartilage disks (Figure 4), creating vibrations of the entire body (Kwong-Brown et al., 2019). Sounds are propagated effectively underwater because of impedance matching; the body is mostly water and the medium is water. *In vivo*, vibrations can be recorded from the entire body, including a single digit. The frog's body thus serves as a “loudspeaker.” This novel mechanism for anuran vocal production allowed *Xenopus* to retain ancestral, terrestrial frog vocal signaling (Feng et al., 2017) during underwater social interactions.

The ability to evoke sex- and species-typical sounds from the *ex vivo Xenopus* larynx (Figure 4) reveals that, unlike mammals and birds, in which respiration paces sound production and the CNS controls sound frequencies via the vocal tract (Matzinger and Fitch, 2021), the spectral features of *Xenopus* vocalizations are intrinsic to the larynx.

**Figure 5:**
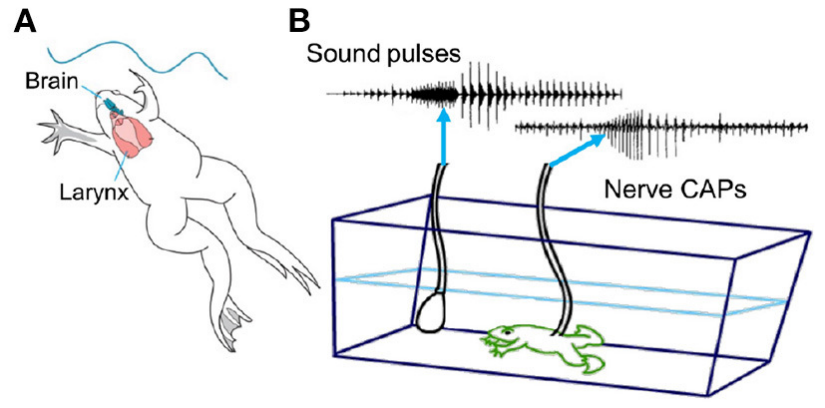
**(A)** The temporal features of species-specific male calls are determined by vocal circuits within the CNS (brain in blue). The spectral features (in Figure 3) are determined by the larynx (in red). **(B)** Each sound pulse is preceded by a compound action potential (CAP) recorded *in vivo* from the laryngeal motor nerve as it enters the muscles posteriorly. Underwater sounds are simultaneously recorded with a hydrophone. Adapted from Yamaguchi and Kelley (2000).

When males sing, neural activity that closely corresponds to actual male and female calls is recorded *en passant* from the laryngeal motor nerve (Yamaguchi and Kelley, 2000; Figure 5). Tightly synchronized Compound Action Potentials (CAPs) recorded from the nerve match the temporal pattern of simultaneously recorded underwater songs across sexes and species (fictive singing: Leininger and Kelley, 2013; Barkan et al., 2018). The temporal features of *Xenopus* songs are generated within the CNS.

## How *Xenopus* hear sounds

**Figure 6:**
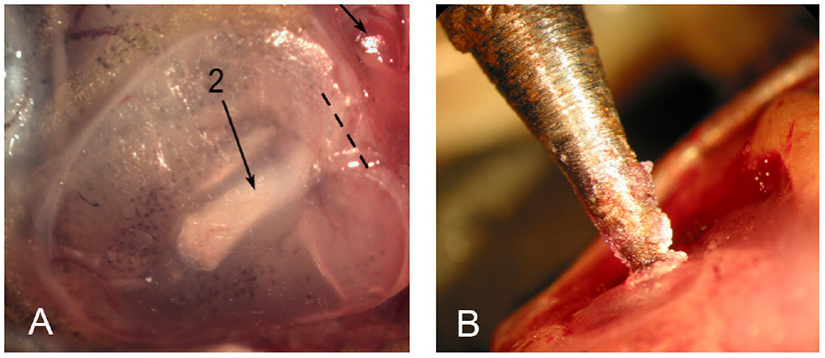
The tympanic disk in *X. laevis*. **(A)** The stapes is visible through the disk (long arrow; 2). Modified from Mason et al. (2009). **(B)** Mechanical vibration delivered to the tympanic disk was matched to actual disk vibrations recorded using laser inferometry in response to underwater calling (Elliott et al., 2007).

Underwater sound waves produce vibrations of the *Xenopus* tympanic disk (Christensen-Dalsgaard and Elepfandt, 1995). The disk is located just behind the eye, under the skin (Figure 6). The stapes (a middle ear bone) inserts into the disk proximally and abuts the oval window distally (Mason et al., 2009). An air-filled cavity connects the two tympanic disks which thus function together as a pressure receiver.

**Figure 7:**
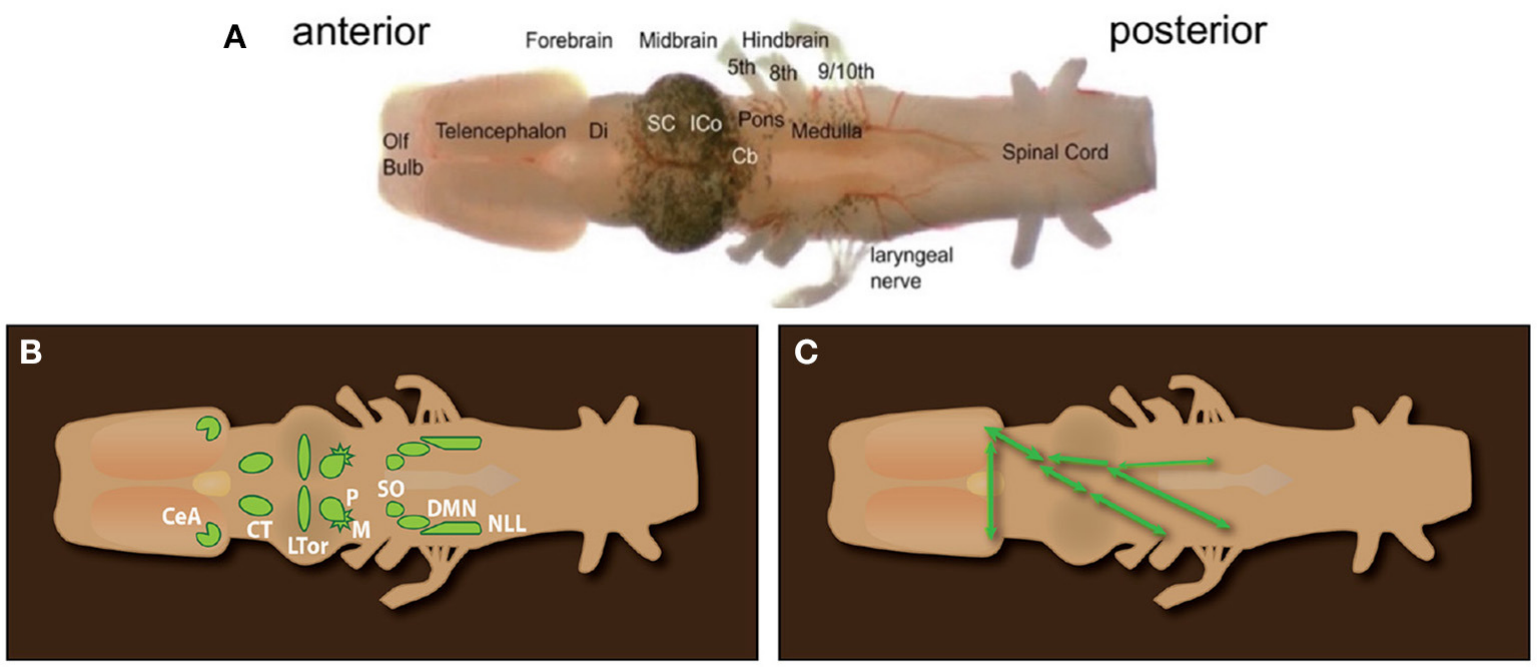
**(A)** *Ex vivo* brain of *Xenopus laevis* from the olfactory bulb (anterior) to the spinal cord (posterior). Cranial nerves 5–9/10 are labeled. Xenopus lacks a tongue as well as the hypoglossal nerve (XII). **(B)** Diagram of octavolateralis nuclei in *X. laevis*. **(C)** Diagrammatic illustration of the connectivity of auditory nuclei. SO to ICo (connections and connectivity in the left auditory pathway not illustrated for clarity). **(A)** Olfactory Bulb, Olf bulb; Di, Diencephalon; thalamus and hypothalamus; SC, Superior Colliculus; i.e., optic tectum; ICo, Inferior Colliculus; Cb, Cerebellum; **(B)** CeA, Central nucleus of the Amygdala; CT, central Thalamus; LTor, Laminar nucleus of the Torus Semicircularis; P, Principal nucleus; M, Magnocellular nucleus.

As in other anurans, the inner ear includes an amphibian and a basilar papilla innervated by fibers of the eighth cranial nerve that arise from neuronal cell bodies in the acoustic ganglion (Homma et al., 2022), and whose terminals innervate post-synaptic neurons in the dorsal medullary nucleus (DMN, Figure 7; Kelley, 1980; Paton et al., 1982). Within the inferior colliculus of the midbrain (ICo), neurons in the laminar nucleus respond to calls (Elliott et al., 2011) and are rate-tuned to temporal properties of specific calls, as is the case for other anurans (Edwards et al., 2007). Song playbacks also activate the nucleus of the lateral line (NLL) and the principal nucleus (P) of the inferior colliculus (Kelley, 1980). Both project to the central nucleus of the thalamus (CT) nucleus (also illustrated below in Figure 7), suggesting that underwater sound waves are also detectable by the lateral line system.

## How the CNS generates *Xenopus* vocal patterns

When serotonin is applied to the isolated brain of males and females (Figure 7A), compound action potentials (CAPS) recorded from the laryngeal nerve (Figure 7A) match male- and female-specific vocal patterns (Rhodes et al., 2007). These patterns are called “fictive calling.” The fast trill portion of the fictive male advertisement call is driven by a rhythmic local field potential produced by neurons in the parabrachial nucleus (PB). The PB is a central pattern generator for advertisement calling.

**Figure 8:**
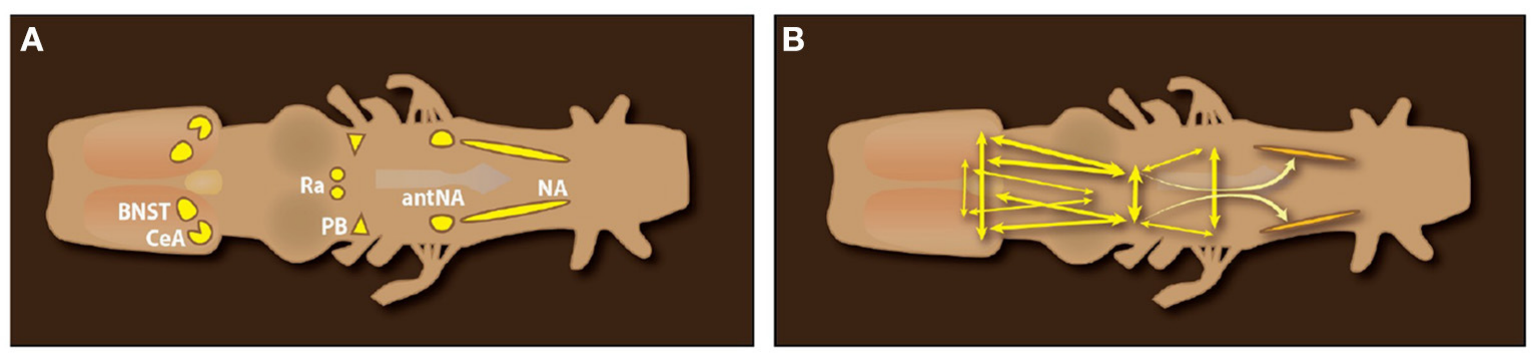
Dorsal view of an *ex vivo Xenopus laevis* CNS. **(A)** Forebrain nuclei implicated in call production: the Central nucleus of the Amygdala (CeA) and Bed Nucleus of the Stria Terminalis (BNST); Midbrain nuclei: the rostral Raphe nucleus pars dorsalis (Ra); Hindbrain nuclei: the pontine parabrachial nucleus (PB), the anterior Nucleus Ambiguus (amNA) and Nucleus Ambiguus (NA) in the medulla. **(B)** Connections of brain nuclei implicated in call production. Double arrowheads indicate reciprocal connections. Connectivity of the Raphe (rRrpd) omitted for clarity. Data from Brahic and Kelley (2003); a candidate homolog of the PAG ventral to the tectum (Figure 10C) was labeled after dye injection into NA.

Anterograde and retrograde mapping in *ex vivo* male and female brains—using fluorescent dextran amines thar travel both anterograde and retrograde—reveal components of the neural circuits that generate vocal patterns (Figure 8). Vocal motor neurons occupy caudal Nucleus Ambiguus, NA. Glottal motor neurons (the glottis is closed during calling), commissural interneurons, and neurons projecting bilaterally to the parabrachial nucleus (PB) occupy anterior Nucleus Ambiguus (antNA). Neurons in PB project throughout NA (shading in Figure 8B), both ipsilaterally and contralaterally, as well as reciprocally. Serotonergic neurons in the rostral Raphe, pars dorsalis (rRpd; Ra in Figure 8) project contralaterally to each other and ipsi- and contralaterally to vocal motor nuclei including the periaqueductal gray (PAG), PB, and NA (Brahic and Kelley, 2003; see also **Figure 10C**). Two forebrain nuclei (the Central nucleus of the Amygdala, CeA) and the Bed Nucleus of the Stria Terminalis (BNST) project to their contralateral counterparts as well as to Ra and PB. The resulting pattern of connectivity (Figure 8B) is highly recurrent and bilateral, insuring effective simultaneous contraction of laryngeal muscles required to produce a sound pulse (Figure 4).

## Reproductive state; Hormones and behavior

In native ponds, the behaviors illustrated in Figure 3—calling and clasping—are seasonal and depend on reproductive state. A sexually reproductive state can be induced in the laboratory by injection of human chorionic gonadotropin (HCG). Embryos can thus be generated at any time of year, greatly facilitating discoveries in developmental, cell and molecular biology (reviewed in Wallingford, 2022) as well as powering discoveries in neurodevelopmental disorders (e.g. Willsey et al., 2021).

The behavioral effects of HCG on male calling are due to gonadotropin itself, to direct effects on neurons expressing gonadotropin receptors in the CeA (Yang et al., 2007), as well as to evoking increased synthesis and release into the circulatory system of gonadal steroids (androgens and estrogens) that activate neurons in the CNS. Sexually unreceptive or ovariectomized females respond to male clasping with leg extension and ticking (Figure 3F) while gonadotropin-injected intact females respond with leg flexion (Figure 3E). Castration abolishes male clasping and calling, behaviors reinstated by androgen treatment (Kelley and Pfaff, 1976; Wetzel and Kelley, 1983). Androgen effects on calling include activating vocal motor neurons as well as their inputs from the parabrachial nucleus. On the auditory side, gonadal hormones effects include direct action on androgen receptor expressing neurons in the acoustic ganglion in the periphery (Kelley, 1981), in the auditory midbrain (Kelley, 1980) and in the CeA of the ventral forebrain, where auditory input and pre-motor output intersect (Hall et al., 2013). Similar patterns of hormone receptor expression are found across other vertebrates (see **Figure 10**).

In summary, innate acoustic communication in *Xenopus* is characterized by species-specificity, a large vocal repertoire, pronounced sexual differences due to secretion of gonadal hormones and male/female, male/male duetting. These features reflect the preeminence of acoustic signals in turbid aquatic habitats over the evolutionary time scales (~ 170 mya, Feng et al., 2017) since the Pipoidae diverged from terrestrial anurans.

## Neural circuit architecture underlying social vocalization in tetrapods

Across phyla (Figure 1), acoustic communication is closely associated with nocturnal species (Chen and Wiens, 2020). Bats, for example, are among the most vocal species, using sound both for locating prey (echolocation) and for social interactions within the roost (Kanwal, 2009). Male sac winged bats (*Saccopteryx bilineta*) produce complex courtship songs directed toward the females in their harems at dawn and dusk, sandwiched between territorial songs (Behr and von Helversen, 2004). The time of day that vocal species are active can vary. Within rodents (~40% of mammalian species), mice (*Mus*) and rats (*Rattus*) are nocturnal while other genera (e.g., *Scotinomys* Neotropical mice), are diurnal (see below). Vocal communication is also prominent in subterranean genera such as naked mole rats (*Heterocephalus glaber*, Credner et al., 1997). Anurans (frogs and toads) are also nocturnal and vocal.

In contrast, birds and humans—perhaps the most highly vocal groups—are predominantly diurnal. Most birds sing as the sun first rises and throughout the day. Even night songsters -i.e., nightingales—join in the dawn chorus (Amrhein et al., 2002). Though humans are diurnal, for most of our evolutionary history, visual cues were not available at night; essential social cues (e.g., your own baby's cry) were vocal. Reflecting diurnal activity, in birds and humans social communication is multimodal; visual cues can shape auditory perception. In humans, watching sound production changes what is heard (the “McGurk effect,” Alsius et al., 2018). In songbirds (zebra finches: *Taeniopygia guttata*), visual signals from conspecifics influence the activity of auditory neurons (George et al., 2011).

As for vocal behaviors, the neural circuits that support acoustic communication in tetrapods leave no trace in the fossil record. We can however compare circuit architectures across vocal vertebrates to determine which features are shared and which are specific to a particular group. For this comparison I've chosen three mammals: a bat (*Pteronotus parnelli*) and two rodents: Alston's singing mice (*Scotinomys teguina*) as well as mice *(Mus musculis*), vocal species with well-characterized repertoires and CNS vocal circuits. As for humans, features of acoustic communication in some species of birds are learned (Jarvis, 2019). Zebra finches and related finches that also learn their songs provide the opportunity to compare circuit motifs across wide phylogenetic distances (Figure 1) as well as providing insight into how acoustic experience and feedback can modify brain circuitry more generally.

### Bats

**Figure 9:**
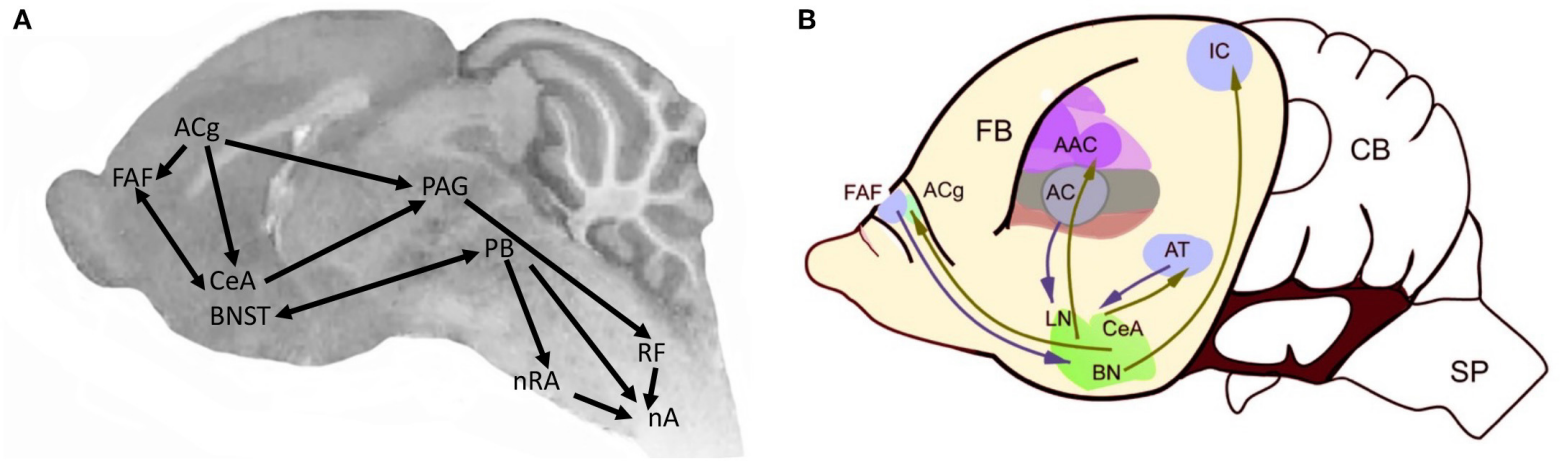
Brain regions associated with vocal communication in *Pteronotus* (modified from Kanwal, 2021). **(A)** The centrobasal amygdala (CBA) includes the CeA (central nucleus) and BN (basolateral nucleus in **(B)**; [modified from Kanwal et al. (2013)]. PAG, periaqueductal gray; PAL, perilemniscal area. Arrow thickness proportional to projection strength. Endocrine regulation is via the hypothalamus (HyTh). AC, auditory cortex; ACg, anterior cingulate; CBA, centrobasal amygdala; FAF, frontal auditory field; nA, nucleus ambiguus; nRA, nucleus retroambiguus; nTS, nucleus of the solitary tract; PAG, periaquedectal gray; PB, parabrachial nucleus; RF, reticular formation. **(B)** Sagittal view of forebrain vocal nuclei in the bat, anterior is to the left. AAC, accessory auditory cortex; AC, auditory cortex; ACg, Anterior cingulate cortex, AT, anterior thalamus; BN, bed nucleus stria terminalis; CB, cerebellum; CeA, central nucleus of the amygdala; FAF, frontal auditory field; FB, forebrain; IC, inferior colliculus; LN, lateral nucleus; SP, spinal cord.

Bats diverged from other Laurasiatherians ~70 mya (Doronina et al., 2017) and comprise ~20% of extant mammalian species. In mustached bats, *Pteronotus parnelli*, adults of both sexes, as well as pups, vocalize during social encounters. Nineteen syllable types are distinguishable acoustically and each is associated with specific social interactions. Ultrasonic vocalizations (USVs), used to locate prey, are also employed during social behaviors. Auditory cortex neurons tuned for echolocation contribute to recognition of USVs used to interact socially at the roost (Washington and Kanwal, 2008). CNS nuclei that support acoustic communication are depicted in Figure 9 (Kanwal et al., 2013; Kanwal, 2021).

CNS vocal circuitry: In *P. parnelli*, mid- and hindbrain neural regions involved in vocal communication include the nucleus ambiguus (laryngeal motor neurons), the reticular formation, the PAG and the PB. Stimulating the CeA evokes agonistic vocalizations (Ma and Kanwal, 2014) and social calls evoke neural activity (Naumann and Kanwal, 2011). Components of the neural circuitry supporting acoustic communication are also responsive to affective and reproductive states (Salles et al., 2019). The distribution of oxytocinergic and vasopressinergic neurons has been mapped (Rao and Kanwal, 2004) and includes forebrain nuclei, such as the CeA. Regions expressing receptors for gonadal hormones such as estrogens and androgens have not been mapped to date. Because expiration drives mammalian vocalizations, in bats that vocalize with open mouths, the activity of muscles such as the diaphragm, the jaw and the tongue must be coordinated, as in *Scotinomys* (see following section) but has not yet been described.

### Rodents

Muroid rodents (rats and mice) comprise ~40% of extant mammalian species and diverged from a common ancestor with lagomorphs ~75 MYA (Churakov et al., 2010). Alston's singing mice, *Scotinomys teguina*, are neotropical, diurnal Cricitine rodents, whose evolutionary divergence was more recent (~7 MYA; Marshall, 1979). As for *Pteronotus*, in all three genera both pup and adult vocalizations are associated with social interactions. A dramatic example in adults is the post-ejaculatory 22 kHz song of male rats (Barfield and Geyer, 1972). Mouse pups produce USV vocalizations when away from the nest that elicit maternal retrieval and both sexes vocalize as the male chases the female before mating (Portfors and Perkel, 2014). In *Scotinomys*, both sexes also vocalize during social interactions associated with mate attraction and competition. Songs are acoustically indistinguishable, although male songs are longer (Banerjee et al., 2019). Pairs of males alternate their songs precisely: “turn taking” [see Vanderhoff and Bernal Hoverud (2022) for a discussion of duetting, turn taking, and antiphony]. When one male is introduced into the cage of another, vocalization is stimulated in both, but always ends with the more variable song of the introduced male (Banerjee et al., 2019).

**Figure 10:**
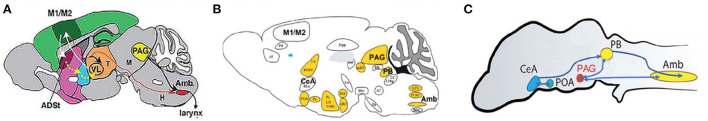
CNS vocal production circuits in sagittal view; anterior is to the left. For mouse **(A)**, Alston's singing mouse **(B)** and *Xenopus* **(C)**. **(A)** *Mus musculis* vocal circuits (adapted from Arriaga and Jarvis, 2013). **(B)** *S. teguina* [adapted from Zheng et al. (2021, 2022); Zhang et al. (2022)]. Nuclei in the vocal circuit identified after pseudorabies injection into laryngeal and jaw muscles. Androgen receptor expression in yellow. **(C)** *Xenopu**s*** (after Brahic and Kelley, 2003, Figure 1B n.III = PAG; Ballagh, 2014). Androgen receptor expression (yellow), estrogen receptor expression (blue); after Kelley (1981). M1/M2, motor cortex; ASDt, anterodorsal striatum; V, trigeminal nucleus; LRF, lateral reticular formation; Amb, nucleus ambiguous; PB, parabrachial nucleus; PAG, periaqueductal gray; POA, preoptic area; LH, lateral hypothalamus; VMH, ventromedial hypothalamus; POA, preoptic area; CeA, central nucleus of the amygdala; BNST, bed nucleus of the stria terminalis; LS, lateral septum.

CNS vocal circuitry: A recent approach to identify brain regions that participate in acoustic communication in mammals is injecting pseudorabies virus (PRV) into vocal muscles and then following transneuronal (retrograde) spread at successive intervals. This PRV approach identifies CNS nuclei that participate in vocal production (Figures 10A,B) and can be combined with monosynaptic anterograde or retrograde tracers to map connectivity (Figure 10A).

*Mice* Arriaga and Jarvis (2013) injected PRV into two laryngeal muscles (CT and CA) resulting (90 h post-PRV injection) in ipsilateral labeling of neurons in Amb (Figure 10A). Injecting BDA into regions of motor cortex—in which neurons express immediate early genes after mice produce USVs—reveals a sparse, apparently monosynaptic, input from M1 onto laryngeal motor neurons (back labeled with a retrograde tracer: cholera toxin). These observations suggest that mouse cortex can directly influence vocal motor neurons.

*Scotinomys* Alston's singing mice (Figure 10B) vocalize with open mouths; movements of jaw muscles must be coordinated with vocal circuits. Injecting PRV into both jaw and laryngeal muscles—and mapping virus-infected neurons up to 96 h post injection—outlines a set of CNS vocal nuclei (Figure 10B) that includes Amb, PB, PAG, CeA, and orofacial motor cortex (OMC). Stimulating OMC in a male during vocal turn taking with another male pauses his song sequence which then resumes at the pause point. Cooling the OMC elongates the song by adding additional notes, slowing song progression. The OMC appears to coordinate male/male singing rather than driving vocal motor production (Okobi Jr et al., 2019). The function of the sparse M1/M2 motor cortex projection to laryngeal motor neurons in mice is not known.

### Anurans

Hindbrain components of CNS circuitry that drive vocal production in frogs (including the PB) were first identified by Schmidt (1976). In *X. laevis*, fluorescent dextran amines applied to the *ex vivo* brain travel both anterograde (labeled fibers and terminal fields) and retrograde (labeled neuronal cell bodies). We used this approach (originally described by Luksch et al., 1996) to identify a projection from the CeA to the pontine parabrachial nucleus (PB: Figure 10C) as well as input to the CeA from auditory thalamus (CT: Figure 7; Hall et al., 2013). In *Xenopus* neurons that drive laryngeal muscles occupy Amb which receives input from the periaqueductal gray (PAG), a brain region recently proposed as a key node for courtship displays across vertebrates (Schwark et al., 2022). The *Xenopus* PB is reciprocally connected to the PAG in the midbrain as well as to the CeA in the forebrain (Figure 10C).

**Figure 11:**
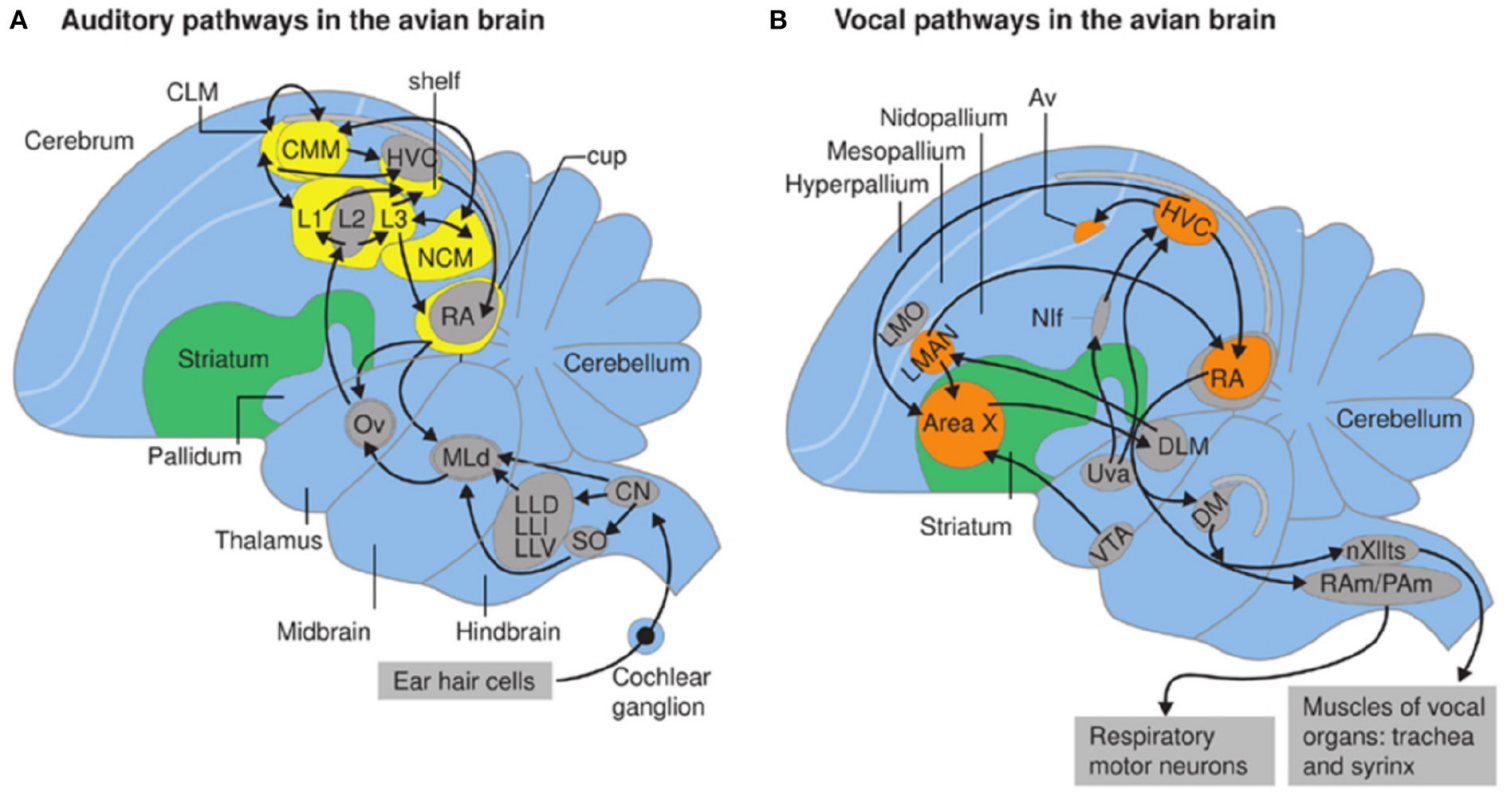
From Berwick et al. (2012). Auditory **(A)** and vocal pathways **(B)** in zebra finch brain; schematic sagittal view; anterior is to the left. **(A,B)** Syringeal motor neurons populate nXIIts (the hypoglossal nucleus, caudal medulla). Respiratory motor neurons in the rostral spinal cord are innervated by nucleus retroambiguus (RAm) and paraambigualis (PAm) that coordinate breathing and vocalizing. **(B)** Forebrain vocal motor nuclei include the higher vocal center (HVC) in the neostriatum which projects to nucleus robustus archstriatalis (RA), a premotor nucleus driving both respiratory and vocal hindbrain neurons (Ram/Pam and nXIII). Nuclei Uva, Nif and AV (Kelley and Nottebohm, 1979; Nottebohm et al., 1982) also provide input to HVc from HVc (YIp et al., 2020). Neurons within nuclei in orange are active while the bird is singing. Nuclei HVc and RA are absent in pigeons but present in species that learn songs (Wild, 1997).

Microstimulation of the CeA in the *ex vivo* brain evokes “fictive calling” in adult males (Hall et al., 2013) as well as females (Ballagh, 2014). The *Xenopus* PAG is reciprocally connected to the PB, identified as the central pattern generator (CPG) for the male advertisement call (Rhodes et al., 2007). PB neurons are intrinsically rhythmically active (Barkan et al., 2018). As the evolution of the cerebral cortex is evolutionarily recent (Striedter and Northcutt, 2019), a projection to the hindbrain vocal circuit is not expected in *Xenopus*. A projection from dorsal forebrain is present in songbirds (Figure 11).

Comparing neural circuit motifs in *Pteronotus* (Figure 9), *Mus, Scotinomys*, and *Xenopus* (Figure 10) reveals shared hindbrain, midbrain and forebrain components of vocal production circuitry, including the nucleus ambiguus, the parabrachial nucleus, the periaqueductal gray and the central nucleus of the amygdala. Conservation of these neural circuit motifs supports an ancient origin for tetrapod vocal circuits.

### Birds

Many birds are accomplished songsters and—in some species—males and females duet (Kingsley et al., 2018; Riebel et al., 2019). Zebra finches (*Taeniopygia guttata*, Figure 1) are the most widely studied species; they are readily bred and maintained in the laboratory as are related species such as Bengalese finches. Both male and female zebra finches produce unlearned vocalizations—calls—to locate other adult conspecifics (e.g., the distance call, Elie and Theunissen, 2018). Within the nest, parent zebra finches employ soft calls to coordinate chick care (Elie et al., 2010). As in other tetrapods, song is powered by expiration (Suthers et al., 1999).

Two aspects of vocal production, however, are avian-specific, presumably reflecting Arcosaur ancestry (Figure 1). While birds have a larynx and vocal tract (including the tongue), the spectral features of their vocalization are shaped by the syrinx, the avian specific vocal organ (Kingsley et al., 2018), (see Albersheim-Carter et al., 2016). In contrast to calls, male courtship songs are learned by young birds in a sequence that resembles human speech acquisition (Thorpe, 1954; Doupe and Kuhl, 1999). Song learning is supported by specialized forebrain nuclei that shape vocal production to reflect auditory experience. Forebrain neural circuits, notably the auditory recipient nucleus (Field L and its subnuclei), as well as the vocal efferent nuclei (HVc and RA, Nif and Av; Figure 11) are not homologs of mammalian auditory and motor cortex (in humans, Wernicke and Broca's area; see Mooney, 2022) although neural circuit motifs do resemble those of mammals (Calabrese and Woolley, 2015). Motifs shared between songbirds with vocal learning and human primates can thus provide insight into convergent principles of neural circuit formation and modifications that support vocal learning.

The bird forebrain auditory-recipient nucleus (Field L in the neostriatum) consists of several interconnected sub-regions (L1-3) whose circuit architecture resembles processing in mammalian auditory cortex (Calabrese and Woolley, 2015), a striking example of evolutionary convergence. Neurons in Field L project to a strip of tissue, originally termed the “shelf” (Kelley and Nottebohm, 1979) immediately ventral to the neostriatal nucleus, HVC (higher vocal center). Axons of HVC neurons travel ventrally to nucleus robustus in the archistriatum (RA) forming an encircling cup (Nottebohm et al., 1982). RA neurons innervate motor neuron pools in the hindbrain that drive respiratory and syringeal muscles. Breaths and “mini-breaths” (brief inspiration bout within a sound-producing expiration) control patterns of vocal expression via expulsion of air from the lungs (Wild et al., 1998), much as in the patterning of cries in mouse pups described below.

Another conserved feature across species with vocal learning is the role of dopamine and the basal ganglia (LMAN and Area X). The young bird “evaluates” the match between a learned song and his own match to that song, linking motor output to its acoustic consequences (Gadagkar et al., 2016; Mooney, 2020). This match is mediated by convergence between inputs to HVc from Nif and midbrain dopaminergic inputs (Tanaka et al., 2018). Dopamine (and the basal ganglia more generally) is implicated in motor patterning across vertebrates (Grillner and Robertson, 2016; Suryanarayana et al., 2022). This widely conserved feature appears to have been exapted for both the modification of vocal circuits essential for language learning in humans and song learning in birds. A recent paper that employed cutaneous sensory stimuli to shape vocal production rather than auditory feedback, also identified a role for dopamine in vocal learning (McGregor et al., 2022). A role for dopamine in reproductive-state dependent odor preferences has recently been identified in *Drosophila* as well (Boehm et al., 2022). Taken together these observations suggest that the ability of dopamine to shape neural circuitry is highly conserved.

## Central pattern generators and vocalization

As discussed below, a vocal CPG that patterns mouse pup cries has recently been identified in the inferior reticular formation (Wei et al., 2022). In *Xenopus*, the parabrachial nucleus (PB) is a CPG for the male advertrisement call [reviewed in Kelley et al. (2020)]. When the *ex vivo Xenopus* brain is exposed to serotonin, fictive advertisement calling CAPs recorded from the laryngeal nerve coincide with a pronounced local field potential recorded from the PB (Rhodes et al., 2007). Transection at various levels of the CNS as well as cooling studies confirm the role of the PB as a vocal CPG. PB neurons retain their intrinsic rhythmicity in the *ex vivo* brain even when isolated synaptically (Barkan and Zornik, 2019). A CPG that drives slow trill has not yet been identified but might correspond to PiCO, a proposed inspiratory CPG in mice (Anderson et al., 2016). While *Xenopus* vocal production is independent of respiration (Figure 4), a rhythmically active neural circuit element that functions to gate the inspiratory/expiratory transition in rats (Dutschmann and Herbert, 2006) functions in *Xenopus* as a CPG controlling vocal rhythms, suggesting exaptation of a respiratory circuit element present in the common ancestor of tetrapods. As discussed below, one class of neurons in the PB, the FTNs, differ intrinsically in rhythmicity across related species, opening a window into genetic divergence that supports speciation (Baker et al., 2019).

As bird songs are coordinated with respiration, one approach to finding a vocal CPG in birds is to identify the respiratory CPG. Wild (1997) described neurons in nucleus retroambiguus (Ram: Figure 11B) projecting to respiratory motor neurons in pigeon and songbirds. RAm efferents were also observed in the PB, rostroventral lateral medulla (RVL), caudal pons and in XIIts. Both RAm and PB have been considered candidate vocal pattern generating nuclei in songbirds. A recent review (Mooney, 2020) suggests instead that the songbird vocal CPG is located in a reticular nucleus, RVL. RVL drives activity of syringeal motor neurons but is gated by neurons in the caudolateral PAG. An alternative suggestion is that RVL coordinates activity of vocal motor neurons (as suggested for LRF in *Scotinomys*) while the homolog of PB contributes controls vocal patterning. If so, the origin of the PB as a vocal CPG could be evolutionarily ancient.

A recent study (Wei et al., 2022) sought to identify a vocal CPG in infant mouse pups by examining the neural circuits that generate USVs. In pups, a single large breath can be associated with either one or multiple cries. For multiples, each cry is accompanied by a smaller increases or decreases in airflow (resembling the “minibreaths” in canary and zebra finch songs, Hartley and Suthers, 1989; Wild et al., 1998). The authors predicted that this vocal pattern is generated by an intrinsically faster CPG that coordinates with the overall breathing pattern. Previous studies in mice have established that breathing is patterned by a inspiratory CPG that includes neurons in the preBotzinger nucleus (PBC). Blocking the activity of laryngeal TA and CT prevented cry production but not the minibreath pattern, suggesting separate CPGs for cry production and minibreaths. Interneurons innervating TA and CT motor neurons form three groups: rv-iRF (glutamatergic neurons), Botzinger and preBotzinger nuclei (gabaergic) and Nucleus Retroambiguus (mixed). Interneurons innervating both tongue motor neurons and TA motor neurons were also found in rv-iRF. Inactivating rv-iRF disrupted the interval between cry bouts as well as intervals within a bout, but not basal breathing. Brief optogentic stimulation of the rv-iRF produced cry bouts throughout the longer breath. Comparing activity patterns in brain slices that included the rv-iRF and the pre-Botzinger nucleus revealed a faster oscillation (every 6s as compared to 23s) in the former. These experiments provide strong evidence that the rv-iRF generates the pattern of pup cries. This vocal CPG provides input to preBotzinger neurons to drive inspiration (triggering minibreaths) and coordinates activity in the laryngeal TA and CT muscles that control glottal opening.

The IRO is also a candidate participant in patterning of the more complex courtship vocalizations of adult mice. At the behavioral level, the overall spectro-temporal features of male mouse USVs develop continuously from pup calls, stabilizing about 4 weeks later. A shared CPG might represent the “common biological mechanism” suggested by Castellucci et al. (2018). Regardless, the Wei et al. study provides an experimental blueprint for identifying candidate CPGs in adult mice as well as other rodents, such as rats and *Scotinomys*. Identification of IRO as a CPG for pup calls does not preclude the participation of other CPGs in patterning vocalizations. In cats, for example, neurons in the parabrachial nucleus (specifically the Kolliker-Fuse nucleus) are rhythmically active during inspiration, post-inspiration and expiration (Dick et al., 1993), providing a candidate vocal CPG (see also Hage, 2010 for discussion of primate vocal CPGs).

## Reproductive state: Comparing CNS gonadal hormone receptor expression across vertebrates

Another highly conserved feature of CNS vocal circuitry is the expression of receptors for gonadal steroids (typically androgen in males and estrogen in females) in auditory and vocal neurons (shown for the androgen receptor in motor components of the vocal circuit in *Scotinomys* (nuclei in yellow: Figure 10B) and for *Xenopus* (nuclei in yellow and green, Figure 10C).

The capacity for synthesizing estrogen arose before the evolution of the ancestral ER (Eick and Thornton, 2011). Steroid hormone receptors are also evolutionarily ancient; derived from a single ancestral receptor that diverged from the nuclear receptor superfamily early in vertebrate evolution. These receptors diversified in the chordates; amphioxus has two: an ER and a member of the AR/PR/GR/MR family. The pipoidae (ancestral to modern pipids including *Xenopus*) emerged during the Jurassic ~170 mya and the genus *Xenopus* ~50 mya (Feng et al., 2017). The Rodentia diverged from a common ancestor with the Lagomorpa ~65 mya (Romanenko et al., 2012) and *Scotinomys* perhaps 7 mya (Fernández-Vargas et al., 2021). While characters related to reproductive signaling (such as sexual dimorphism) can be lost as well as gained evolutionarily (e.g., Leininger and Kelley, 2013), similarities in the distribution of androgen receptors in two evolutionarily very distant species (*Xenopus* and *Scotinomys*, see below) suggest an ancient role in the coordination of vocal signaling during reproduction.

*Xenopus* In *X. laevis*, androgen (acting synergistically with gonadotropin) controls male clasping (Kelley and Pfaff, 1976) and estrogen (acting synergistically with LHRH and gonadotropin) controls female receptivity (Kelley, 1982). Gonadectomy abolishes adult reproductive behaviors in both sexes. These hormones also participate in the control of vocal communication. Gonadal steroid receptors are expressed in brain regions implicated in acoustic communication; from the acoustic ganglion through to the larynx during both development and adulthood including laryngeal motor neurons (Kelley, 1980).

In females, but not in males, preferential auditory evoked potential responses to each species' dominant frequencies are abolished by ovariectomy and reinstated by androgen (Hall et al., 2013). Testosterone is the major circulating gonadal steroid in female *Xenopus* (Lutz et al., 2001) but can be converted to estrogen *in situ* by aromatase. Neurons within the CeA express estrogen (Figure 10C) and gonadotropin receptors (Yang et al., 2007). Gonadotropin synergizes with androgen to restore calling to castrated makes (Wetzel and Kelley, 1983). The CeA receives auditory input and is required for males to produce socially appropriate responses to female calls (Hall et al., 2013). Laryngeal motor neurons in NA express androgen receptor (Kelley, 1980). The vocal pattern generator (PB) includes neurons expressing androgen but not estrogen receptor (Figure 10C).

*Bats* as Kanwal points out: “There is a deep connection between hormones, the perception and production of social vocalizations, and behavior. Hormones-to-circuits-to-perception or production is a bi-directional process… hormones can modulate and set up either transient or long-lasting neural circuits for the processing, perception, and production of sounds, particularly those having social consequences” (Kanwal, 2021, p. 239). Neurons in the DSCF (Doppler-shifted constant frequency) region of *P. parnelli* respond both to echolocation and to social vocalizations (Washington and Kanwal, 2008) and processing is lateralized in males (but not females) with more responsive neurons in the left hemisphere (Kanwal, 2021), suggesting a sex difference likely to be driven by gonadal hormones. While the locations of gonadal hormone receptor expressing neurons have not yet been mapped, the bat CeA most likely shares this common vertebrate circuit motif. Current research on bat social communication is shifting to *Carollia perspicillata*, as this species is more readily maintained in breeding colonies and uses complex vocal interactions to communicate. Individual *C. perspicillata* have distinctive vocal signatures. Distress calls have been shown to activate neurons in the amygdala (Hechavarría et al., 2020). Mapping gonadal steroid hormone receptor distributions in *C. perspicillata* will be a useful test of evolutionary hypotheses.

*Singing mice* despite evolutionary divergence (Figure 1) circuit motifs for acoustic communication and AR expression in *S. teguina* share multiple features with other tetrapods, inclding *Xenopus* (Figure 11). Notably, in both species, vocal motor neurons in nucleus ambiguus and pre-motor neurons in the parabrachial nucleus express androgen receptor (yellow B; yellow and green, C). Neurons in the inferior colliculus of both species also express AR (not illustrated in B). While estrogen receptor expression has not been mapped in *Scotinomys*, ER is expressed in inferior colliculus and CeA of laboratory mice (Charitidi and Canlon, 2010) and is likely to also be expressed in auditory nuclei of singing mice.

*Song birds* As for anurans and rodents, androgen receptor expression is widespread in bird vocal control nuclei (Figure 11B); estrogen receptor however is limited to HVc (Frankl-Vilches and Gahr, 2018). In female white-crowned sparrows, circulating estrogen during the breeding season increases responses of auditory neurons in Field L (Figure 10A) (Caras et al., 2012) as well as immediate early gene expression in the social behavior network (Maney et al., 2008). Auditory responses to song have also been recorded in the bird homolog of the amygdala, nucleus taenia (Fujii et al., 2016). Hormonal regulation of both sensory and motor neural circuits that participate in vocal courtship in vertebrates appears ancient.

## The CeA: A conserved node for vocal communication across vertebrates

The central nucleus of the amygdala (CeA) has been described as the “autonomic” amygdala because of its role in respiration and heart rate. Given the prominence of expiration for vocal expression across vertebrates, CeA involvement in vocal communication makes sense. In *Pteronotus* CeA stimulation evokes agonistic vocalizations (Ma and Kanwal, 2014). Neurons in the CeA also respond to social vocalizations, especially those associated with aggression. In primates, a baby's cry activates the parents' amygdala (Riem et al., 2021). Autonomic rhythms pace vocalizations of marmosets (Zhang and Ghazanfar, 2016). Output from the CeA (central-medial boundary) to the PAG transiently suppress vocalization in mouse pups (Tschida et al., 2019). In adult mice, activating neurons in the preoptic area of the hypothalamus (POA) that express estrogen receptor in adults inhibits inhibitory PAG neurons allowing USV expression as well as scaling the duration and persistence of bouts (Chen et al., 2021).

In *Xenopus*, a species that uncoupled breathing from calling many millions of years ago, the CeA matches acoustic stimuli to vocal expression. Lesions of the CeA in *Xenopus* result in socially inappropriate responses of males to song playbacks (Hall et al., 2013). Lesioned males respond to broadcasts of rapping and even an actual rapping female (Figure 3G) with prolonged vocal suppression (the response normally elicited by a vocally dominant male) rather than answer calling (the socially appropriate response; Figure 3). In *Xenopus*, the inhibitory output from CeA to a putative PAG homolog is conserved. However, unlike mammals, the *Xenopus* APOA does not project to PAG directly, instead innervating and receiving input from rRpd (Brahic and Kelley, 2003). rRpd, a serotonergic nucleus, is reciprocally connected to APOA, PB and NA and also projects to PAG. Thus, while in mice POA ER-expressing neurons have direct access to the PAG—inhibiting inhibitory neurons and promoting vocalization—in *Xenopus*, APOA neurons may influence vocalization via serotonergic innervation of PB and NA.

## Neural circuit motifs that generate species-specific vocal rhythms; Genetic approaches in *Xenopus*

**Figure 12:**
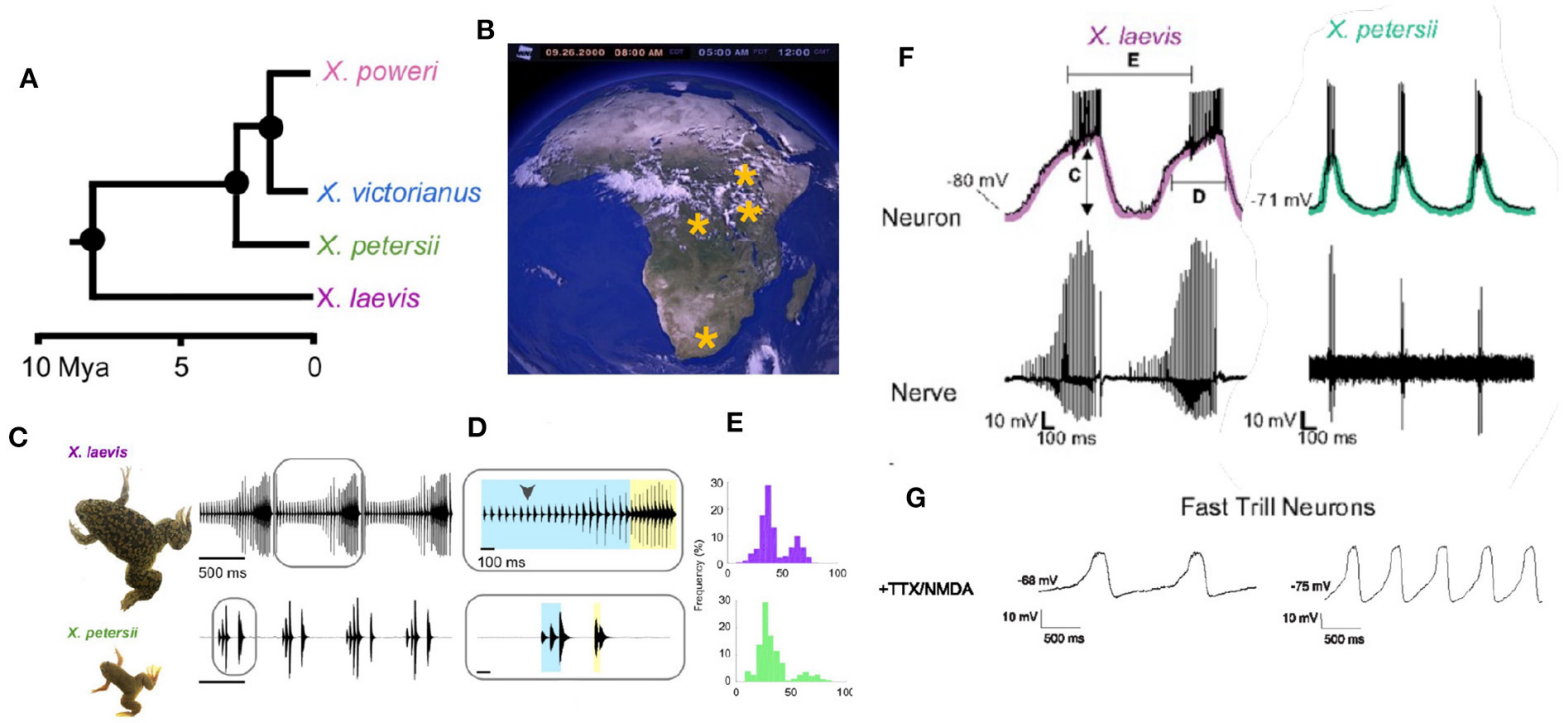
**(A)** Evolutionary divergence within the L subgroup. **(B)** Geographic distributions of L clade species across Africa. **(C–E)** *X. laevis* and *X. petersii* both produce biphasic calls. **(F)** In both species, a sub-set of PB neurons (Fast Trill Neurons or FTNS) spike rhythmically at the onset of fast trill (category defined by measuring **C–E**). **(G)** When synaptically-isolated and exposed to NMDA, the membrane potential of FTNs oscillates at the species-specific rhythm of the advertisement call. Modified from Barkan et al. (2018).

The persistence of species depends on successful reproduction: the production and survival of offspring that go on to reproduce and survive themselves (Darwin, 1872). Because hybrid offspring can be disadvantaged in development, survival and/or mating, identifying a potential mate of the same species is a primary imperative (Lemmon and Lemmon, 2010). As there are 29 extant species of *Xenopus*, in all of which males produce advertisement calls (Tobias et al., 2011), evolutionary conservation of neural circuitry across the genus can be explored. For example, the role of the PB in generating vocal patterns in *Xenopus* was evaluated recently by cross-species comparison between *X. laevis* and *X. petersii*, members of the L species subgroup that diverged ~8 mya (Figure 12A).

A specific class of rhythmically active neurons (Fast Trill Neurons or FTNs) in the PB was identified electrophysiologically in both species (Figure 12F). When synaptically- isolated by blocking sodium channels and stimulated by application of a glutamate agonist (NMDA), the membrane potential of FTNs oscillates at the species-specific rate and rhythm. This inter-species observation strengthens the identification of the PB as the vocal CPG (Rhodes et al., 2007) and implicates a specific class of rhythmically active neurons in divergence of vocal signaling across the L subgroup.

To drive the beginning phases of speciation that resulted, for example, in the different advertisement calls of the L subgroup, divergence in male courtship songs across populations must have co-evolved with female sensitivity to—or preference for -acoustic features of those songs (or *vice versa*). In *Xenopus*, each sound pulse includes two dominant frequencies (Figure 1) that differ across species. In the L subgroup, the DF2/DF1 ratio is 1.22, except for *X. laevis* in which the ratio is 1.14 (Kwong-Brown et al., 2019). Auditory evoked potentials reveal that females are preferentially acoustically sensitive to species-specific DFs at the species-specific ratio (Hall et al., 2013), suggesting that this spectral feature is salient for same species recognition by females in L subgroup species. Both temporal and spectral features of male and female calls determine vocal responses in *X. laevis* (Vignal and Kelley, 2007). As described above, in most vocal vertebrates the CNS controls both the temporal features of songs (via respiratory/vocal CPGs) and song spectral features (via hypoglossal control of the vocal tract). In *Xenopus* however the brain controls only the temporal features while spectral features are inherent to the larynx. This separation simplifies the genetic analysis of song divergence during speciation.

Within the L subgroup, advertisement calls are species-specific. What differences in gene expression between FTNs in the hindbrain and the vocal organ of different species contribute to species specific vocal signlling? Unusually, in *Xenopus* interspecific hybrids between extant species can produce fertile F1 and F2 offspring of both sexes (Evans, 2008). Genetic candidates for speciation-associated divergence in song temporal features are loci encoding or modulating FTN ion channels (Barkan et al., 2018). Candidates for species divergence in song spectral features are loci that contribute to the laryngeal cartilage components that support production of the two dominant frequencies (Kwong-Brown et al., 2019). To function in species divergence, female vocal perception and preference and diverging male songs must co-evolve. Recent research in two invertebrates, Hawaiian crickets and fruit flies, is revealing genetic architectures that support co-ordination of evolution of acoustic signaling in the sexes.

## Neural circuit motifs that generate species-specific acoustic communication: Invertebrates

### Crickets

Crickets use acoustic communication at a distance (far field) during courtship. Interestingly, female preferences co-evolve with acoustic features of male courtship songs in Hawaiian crickets (*Laupala*) (Xu and Shaw, 2021). Many small to moderate effect genetic loci are linked to species differences in male pulse rates. Fine mapping using high density SNP linkage maps has narrowed QTL confidence intervals and permitted annotation of genes within QTL peaks, highlighting candidate genes for linked production and preference. Comparison of species pairs from different islands revealed that, despite the many small to moderate effect sizes, multiple interspecific divergences of *Laupala* mating songs involve similar genetic architectures and share more QTL than were expected. Notably, pulse rate (male) and pulse preference (female) co-localize in the genome, raising the possibility that the linkage between male performance and female preference contributes to shared QTL.

QTL in *Laupala* are associated with genomic regions that—in the fruit fly *Drosophila*—are associated with neuronal development, rhythmic action and neuromodulators known to influence CPGs (Blankers et al., 2018). Combining the cellular approaches to production and perception of songs in field crickets (*Grillus;* Schöneich, 2020) with recent approaches to cricket genome editing and manipulation (Nakamura et al., 2022) should provide an experimental arena for testing candidate genes from QTL fine mapping.

### Fruitflies

**Figure 13:**
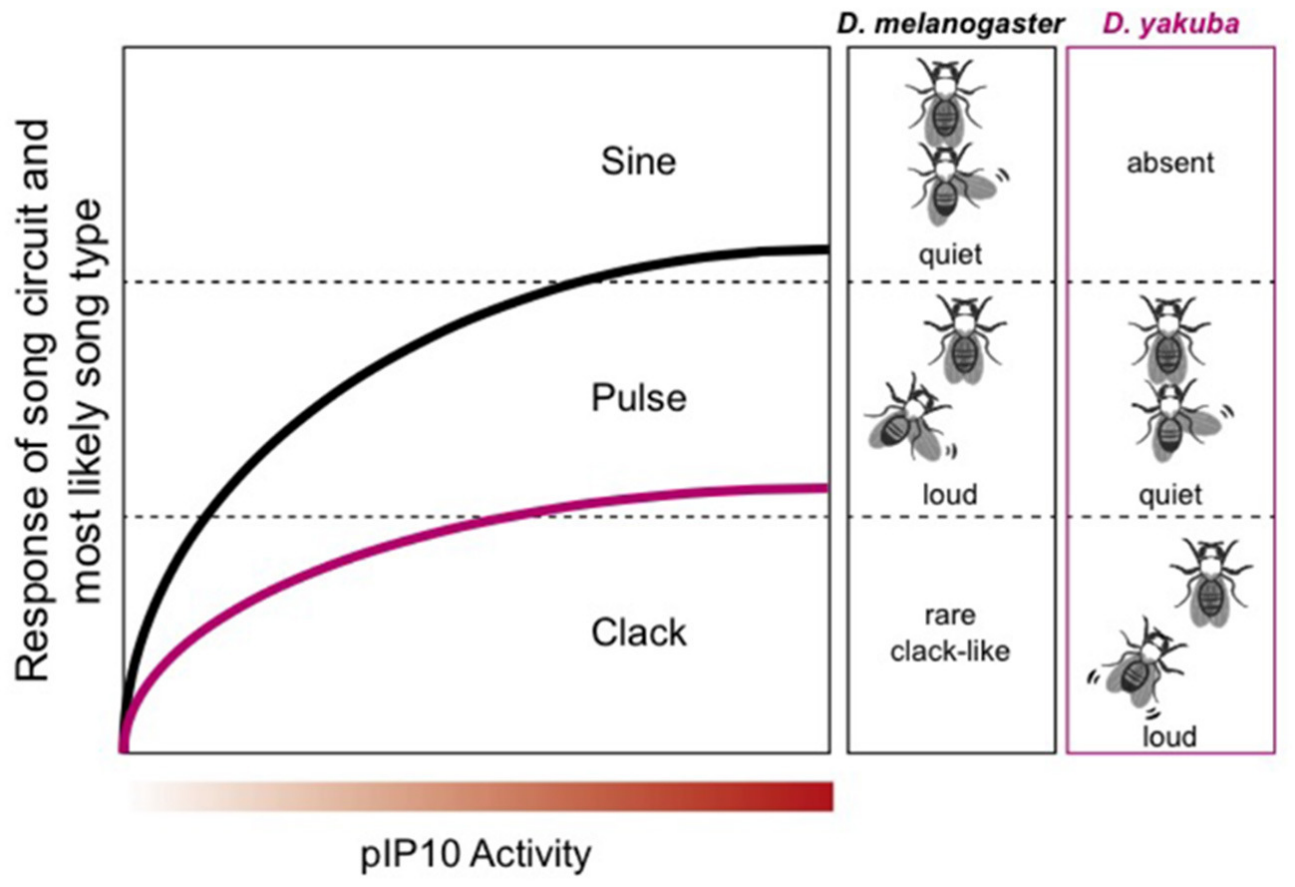
Modified from Ding et al. (2019). In *D. melanogaster*, male song consists of two phases: sine and pulse. Pulse song, which is intense, dominates when the male is chasing the female. *D. yakuba* uses the “clack” song while chasing the female. Male *D. melanogaster* sometimes—but rarely—produce a clack song in response to plP10 activation. Thus, whatever neural machinery is necessary to produce a clack song that machinery is present in both species.

Fruitflies use acoustic communication during courtship (Murthy, 2010). Male songs are generated by wing vibration and neural circuitry supporting species-specific acoustic communication has been mapped in detail for *D. melanogaster* (reviewed in Sato et al., 2020). The *Drosophila melanogaster* subgroup includes 9 species with evolutionary divergence times (relative to *melanogaster)* ranging from ~5 (*simulans)* to ~13 mya (*yakuba*: Tamura et al., 2004). A recent study identified a homologous descending interneuron in *D. melanogaster* and *D. yakuba* (plP10) that is activated by similar social contexts, but drives different motor outputs (Ding et al., 2019). In both species, louder songs are used while chasing females (pulse for *D. melanogaster* and cluck for *D. yakuba*). *D. melanogaster* can produce clack songs, suggesting that circuitry for this song type is shared between species (Figure 13).

### Comparing vertebrate and invertebrate sound communication

Because plP10 can drive both “clack-like” and “pulse” song; it can be considered a “multipurpose” interneuron, with access to at least two motor programs (Figure 13). *Drosophila melanogaster* and *yakuba* plP10 neurons are electrophysiologically similar (Ding et al., 2019). The species difference is the intensity with which a defined interneuron (plP10) drives downstream song production. Low levels of plP10 activity drive high amplitude songs and high levels of activity drive low amplitude songs (Figure 13). Inhibitory sound control circuitry must be interposed between plP10 and motor neurons involved in the wing vibrations that produce songs. Disinhibition is also a circuit motif in mice. USVs can be elicited by stimulating ER1+ neurons in the LPOA, relieving an inhibitory clamp in the PAG (Chen et al., 2021). Increasing the intensity of stimulation in mice scales USV intensity and duration, although in a direction opposite to *D. melanogaster*. In *X. laevis*, microstimulation of the CeA in the *ex vivo* brain drives fictive singing (Hall et al., 2013). The CeA is almost entirely GABA-ergic (Brox et al., 2003) thus, as in *D. melanogaster* and mice, disinhibition (gating) is a prominent circuit motif. In mouse pups, the output of the CeA to the PAG is also inhibitory and disinhibition gates production of their cries (Tschida et al., 2019). Disinhibition thus gates acoustic communication across phyla.

For *Xenopus laevis* and *petersii*, as in *D. melanogaster* and *yakuba*, the species difference is apparent in interneurons rather than, for example, sensory or motor neurons. *Xenopus* FTNs display species-specific electrophysiological properties: cell autonomous, species-specific membrane oscillation rhythms when stimulated with NMDA (Figure 12). PB neurons provide high fidelity, excitatory innervation directly to laryngeal motor neurons (Zornik and Kelley, 2008). In *Xenopus*, motor neurons modulate CPG activity (“feedback to the future”; Barkan and Zornik, 2019). Axon collaterals from laryngeal motor neurons synapse on inhibitory interneurons that control the precision (i.e., the interval between spikes that ride on each PB neuron oscillation) with which PB drives the vocal pattern. Whether this circuit motif for vocal precision occurs in other species remains to be determined. Retuning the PB CPG fast trill neurons across species might seem analogous to differences in the output of plP10. However, *Xenopus* FTNs provide monosynaptic excitatory input that drives vocal motor neurons while the *Drosophila* plP10 is an inhibitory synapse onto a downstream, interneuronal circuit motif.

### Matching production and perception/preference

A still mysterious aspect of the divergence in vocal communication that accompanies speciation—in both vertebrates and invertebrates—is how perception or preference of the receiver for an acoustic signal—and the production of that signal—co-evolve (see Yeh, 2022, for a recent example in zebra finches). Matching production and perception during speciation is not confined to vocal signaling. Sensory stimuli associated with a non-reproductive benefit—such as a specific color that signals a desirable food—might be adopted to create or enhance attractive signaling: the “sensory trap” and “sensory exploitation” hypotheses [reviewed in Ryan (2021)]. However, neither hypothesis directly addresses how the production of communication signals and the acoustic recognition of those signals co-evolve as species diverge, at the level of underlying neural circuit functions.

In *Xenopus*, vocal production is supported by a dedicated CNS motor pathway and neuromuscular control of contractions of laryngeal muscles. Species-specificity reflects the intrinsic patterned activity of FTN neurons in the PB. Vocal perception is influenced both by detectability and recognition. Neurons in the acoustic ganglion of females support enhanced detectability of own-species sound pulse dyads (Hall et al., 2013). Neurons in the anuran auditory midbrain (ICo) are tuned to sound pulse rate, supporting recognition both for call type within a species (Figure 3) and potentially for recognizing conspecifics. Reproductive state gates vocal communication in both sexes via expression of receptors for gonadal hormones acting on the vocal communication system from the level of primary auditory neurons to vocal muscles.

Speciation in *Xenopus* follows two trajectories. One occurs in the L and M clades (tetraploid species with different call patterns: L; biphasic and burst; M: burst and click, Tobias et al., 2011) suggesting evolutionary divergence driven by sexual or natural selection, rather than genetic drift. *Xenopus* also speciate by hybridization, resulting in genome sizes ranging from tetraploid to dodecaploid (A species group). The A group is the most speciose in the genus and the female release call (ticking, Figure 3) is absent (Tobias et al., 2014) suggesting the possibility that loss of the female unreceptive call facilitated hybridization. As ticking can also be produced by the *ex vivo* brain of female *X. laevis* this preparation could be very useful in figuring out the basis—neural circuit and genetic architecture—for the loss of ticking in the A species group.

In other frogs, recognizing a heterospecific male is selected for in females because F1 hybrids are less fit. In *Pseudacris*, for example, the lifetime fitness of hybrid males, but not females, is reduced by 44% (Lemmon and Lemmon, 2010). The simplest hypothesis for co-evolution of vocal signaling in males, and preference in females, is overlapping gene networks in neurons that produce and respond to sounds. Lemmon and her colleagues (Ospina et al., 2021) compared divergence of gene networks in populations of *P. ferriarum* in sympatry or allopatry with *P. negrita*. They identified seven candidate synaptic transmission genes that have diverged between these populations, with more genes overall diverged between females than males. Neurons in the anuran inferior colliculus are selectively driven by interpulse interval (Edwards et al., 2007). Preliminary studies suggest differences in tuning of *Pseudacris* ICo neurons between sympatric and allopatric populations, providing a possible neural substrate for matching production and perception. Whether this difference is sex specific (reflecting greater genetic divergence in females) is not yet clear but if so, could be due to gonadal hormones.

## Multimodal signaling, sex, speciation and language

Acoustic signaling is ancient and phylogenetically associated with extant species that are nocturnal or especially vocal at dawn or dusk (Figure 1, Chen and Wiens, 2020). Birds and humans are the major exceptions. Humans display the “McGurk effect” in which visual stimuli from the face of a speaker influence the acoustic identification of a syllable (McGurk and MacDonald, 1976). In starlings, conspecific visual stimuli also modify responses of neurons in the primary forebrain projection area—Field L—to familiar and unfamiliar songs: familiar songs suppress responses and unfamiliar songs enhance responses (George et al., 2011). Multimodal sensory integration is thus also likely to have shaped the evolution of vocal communication in diurnal species such as primates and birds.

Because speciation reflects both sexual selection (success in attracting mates) and natural selection (survival), sex and speciation are linked at many levels. In vertebrates, sexual differentiation is governed by pituitary and gonadal hormones. Patterns of AR and ER expression—from sensory receptors through to neural circuits for muscle effectors—are targets for evolutionary selection. Broder et al. (2021) argue that “it may be easier than assumed to evolve new sexual signals because sexual signals may be arbitrary, sexual conflict is common and receivers are capable of perceiving much more of the world than just existing sexual signals.” Part of this argument is based on the idea that an arbitrary sensory stimulus associated with a positive experience (for example a red food source) can be co-opted to shape behaviors, such as approach (sensory exploitation). However, such co-opted sensory stimuli are not necessarily useful finding or selecting reproductive partner of the same species for reproduction. This is particularly for true females whose gametes are finite and provide resources for the embryo, unlike those of males.

Robert and the late Dorothy Cheyney argued (Seyfarth and Cheney, 2014)—using multi-year field data on vocal communication in baboons—that the origin of human language might lie in social cognition. Baboons have a matrilineal dominance hierarchy; each female has a distinctive “grunt” vocalization. Seyfarth and Cheyney recorded vocalizations during social interactions between all female pairs in Year 1. In Year 2, they observed female A/female B interactions and then played back A's call to B to determine whether B's response to the playback reflected what had happened (grooming, for example, or biting) during that specific interaction. Did B stay put (grooming: positive interaction) or move away (biting: negative interaction)? They reported that B's response was triggered specifically by A's grunt and matched the social valence of their recent interaction. If indeed the substrate for language evolution, we have much more to learn about the neurobiology of vocal communication across species.

While humans do not actually bite each other during arguments (at least as adults), we do use biting language. We also devote considerable attention to decoding how people feel about us from cues in voice to construct a socially appropriate response. Areas of the human brain involved in language production and perception must (at the very least) access other areas that identify social context-driven voice cues regulated by the endocrine and neuromodulatory systems described in this review. Advances in fMRI now allow imaging of entire brains in response to conspecific and heterospecific vocal sounds in other animals (Van Ruijssevelt et al., 2013; Gábor et al., 2020). Imaging whole brain activity during vocal communication across species over the next few years will drive additional discoveries in this scientific arena.

## Data availability statement

The original contributions presented in the study are included in the article/supplementary material, further inquiries can be directed to the corresponding author.

## Author contributions

DBK wrote this manuscript and adapted from published work or created the figures.

## Conflict of interest

The author declares that the research was conducted in the absence of any commercial or financial relationships that could be construed as a potential conflict of interest.

## Publisher's note

All claims expressed in this article are solely those of the authors and do not necessarily represent those of their affiliated organizations, or those of the publisher, the editors and the reviewers. Any product that may be evaluated in this article, or claim that may be made by its manufacturer, is not guaranteed or endorsed by the publisher.
